# Intestinal and Hepatic Uptake of Dietary Peroxidized Lipids and Their Decomposition Products, and Their Subsequent Effects on Apolipoprotein A1 and Paraoxonase1

**DOI:** 10.3390/antiox10081258

**Published:** 2021-08-06

**Authors:** Xueting Jiang, Pragney Deme, Rajat Gupta, Dmitry Litvinov, Kathryn Burge, Sampath Parthasarathy, Chandrakala Aluganti Narasimhulu

**Affiliations:** 1 Burnett School of Biomedical Sciences, College of Medicine, University of Central Florida, Orlando, FL 32827, USA; jiangxt@igwfmc.com (X.J.); pdeme1@jhmi.edu (P.D.); rajatgupta@ucla.edu (R.G.); DLitvinov@iab.ac.ru (D.L.); spartha@ucf.edu (S.P.); 2 Department of Pediatrics, Division of Neonatology, University of Oklahoma Health Sciences Center, Oklahoma City, OK 73104, USA; kathryn-burge@ouhsc.edu

**Keywords:** oxidized lipids, linoleic acid, free fatty acid peroxides, oxononanoic acid, azelaic acid, paraoxonase 1, ApoA1

## Abstract

Both pro- and antiatherosclerotic effects have been ascribed to dietary peroxidized lipids. Confusion on the role of peroxidized lipids in atherosclerotic cardiovascular disease is punctuated by a lack of understanding regarding the metabolic fate and potential physiological effects of dietary peroxidized lipids and their decomposition products. This study sought to determine the metabolic fate and physiological ramifications of 13-hydroperoxyoctadecadienoic acid (13-HPODE) and 13-HODE (13-hydroxyoctadecadienoic acid) supplementation in intestinal and hepatic cell lines, as well as any effects resulting from 13-HPODE or 13-HODE degradation products. In the presence of Caco-2 cells, 13-HPODE was rapidly reduced to 13-HODE. Upon entering the cell, 13-HODE appears to undergo decomposition, followed by esterification. Moreover, 13-HPODE undergoes autodecomposition to produce aldehydes such as 9-oxononanoic acid (9-ONA). Results indicate that 9-ONA was oxidized to azelaic acid (AzA) rapidly in cell culture media, but AzA was poorly absorbed by intestinal cells and remained detectable in cell culture media for up to 18 h. An increased apolipoprotein A1 (ApoA1) secretion was observed in Caco-2 cells in the presence of 13-HPODE, 9-ONA, and AzA, whereas such induction was not observed in HepG2 cells. However, 13-HPODE treatments suppressed paraoxonase 1 (PON1) activity, suggesting the induction of ApoA1 secretion by 13-HPODE may not represent functional high-density lipoprotein (HDL) capable of reducing oxidative stress. Alternatively, AzA induced both ApoA1 secretion and PON1 activity while suppressing ApoB secretion in differentiated Caco-2 cells but not in HepG2. These results suggest oxidation of 9-ONA to AzA might be an important phenomenon, resulting in the accumulation of potentially beneficial dietary peroxidized lipid-derived aldehydes.

## 1. Introduction

Epidemiological evidence has suggested a relationship between high-fat diets, especially diets high in saturated fat [1], and the incidence of coronary artery disease (CAD) [2,3,4]. The Western diet is rich in fats and contains high levels of peroxidized fats [5,6,7]. Deep-fried foods, and the culinary oils in which they are cooked, contain high levels of potentially harmful peroxidized lipids and their decomposition products [6]. Peroxidized lipids have been shown to induce oxidative stress, generate toxic aldehydes, and perturb biological molecules and membranes [8,9,10,11,12,13,14,15]. The pathophysiological effects of these lipids have been documented extensively in CAD [12,13,14,16,17] and cancer [15], in addition to other contexts [18,19,20].

As the most common polyunsaturated fatty acid (PUFA) in plants and a major component of the vegetable cooking oils heavily utilized in the Western diet, linoleic acid (LA) is likely the largest substrate pool for PUFA peroxidation in the daily diet [21]. While LA cannot be directly synthesized in the mammalian system, humans consume large quantities of linoleic acid via dietary products, such as red meat, nuts and seeds, eggs, and the aforementioned vegetable oils [22]. Two major peroxide products of LA are 13-hydroperoxyoctadecadienoic acid (13-HPODE) and 13-hydroxyoctadecadienoic acid (13-HODE). As peroxides are chemically unstable, 13-HPODE decomposes further to generate 4-hydroxynonenal (4-HNE) and 9-oxononanoic acid (9-ONA) [23]. As several studies have demonstrated the utility of 4-HNE as a biomarker for disease, these decomposition products of 13-HPODE are of specific interest [24,25]. Unlike 4-HNE, the physiological role of 9-ONA has been poorly studied. In a mouse model of CAD utilizing a cholesterol-rich diet, pure forms of fatty acid peroxides accelerated the development of atherosclerosis [26]. On the other hand, peroxidized fatty acids have also been shown to increase the expression of apolipoprotein A1 (ApoA1), a component of the lipoprotein complex generally believed to provide some level of protection against atherosclerosis, high-density lipoprotein (HDL) [27]. A lack of understanding surrounds the apparent opposing roles of fatty acid peroxides in atherosclerosis.

Mammalian ApoA1, the primary protein component in HDL [28,29,30], is synthesized principally in the small intestine and liver [31,32]. Levels of ApoA1 are positively correlated with HDL-cholesterol (HDL-C) and negatively correlated with atherosclerotic cardiovascular disease [33,34,35,36]. The cardioprotective effects of HDL have been largely attributed to the ability of ApoA1 to initiate cholesterol efflux, thereby facilitating the removal of excess cholesterol from peripheral tissues and delivering it to the liver for degradation through reverse cholesterol transport (RCT) pathways [37,38,39,40]. In addition to their roles in reverse cholesterol transport, ApoA1 and HDL are implicated in preventing the oxidative modification of low-density lipoprotein (LDL) and removing lipid peroxides or oxidatively modified lipids from modified LDL [41,42,43]. Importantly, lipid peroxidation products have been shown to induce ApoA1 synthesis [44].

Apolipoprotein B (ApoB), the predominant structural protein of chylomicrons, very low-density lipoprotein (VLDL), intermediate-density lipoprotein (IDL), and LDL, exists in the isoforms ApoB48 and ApoB100 [45]. ApoB48 is synthesized exclusively by the small intestine as a component of chylomicrons [45]. Alternatively, ApoB100 is produced in the liver during VLDL synthesis. These two lipoproteins, both positively correlated with atherosclerotic cardiovascular disease [46], are encoded by the same gene and share the same N-terminal sequence [45]. Peroxidized lipids incorporated into small intestinal chylomicrons may be repackaged during liver synthesis of VLDL [47], providing a potential source of dietary peroxidized lipids within the liver.

Our previous study revealed that oxidized free fatty acids (Ox-FFAs), including 13-HPODE and 13-HODE, are efficiently taken up by differentiated Caco-2 colonocytes with fully mature and intact brush borders [48]. Due to the potential effects of Ox-FFA on the induction of ApoA1 synthesis, and more generally, their role in the atherosclerotic process, the fate of Ox-FFA following interaction with both the intestinal epithelium and liver is of interest. However, Ox-FFAs are subject to autodegradation [49]; thus, the potential effects of Ox-FFAs or their subsequent decomposition products on intestinal and hepatic ApoA1 synthesis and secretion are unknown. In addition, whether the effects of 13-HPODE and 13-HODE uptake differ in the small intestine and liver has not been explored. Here, we determined the metabolic fates of 13-HPODE and 13-HODE, and their decomposition products, following uptake by both immature and fully differentiated intestinal Caco-2 cells, as well as the hepatic HepG2 cell line. This is the first study to address the metabolic fate of ONA, the 13-HPODE decomposition product derived from the carboxylic end of the Ox-FFA, following uptake in the intestine, using a combination of radioactivity, mass spectrometry, and traditional gene and protein expression. Understanding the consequences of intestinal and hepatic interaction with dietary peroxidized lipids may provide light on the apparent duality of roles in the context of atherosclerotic cardiovascular disease.

## 2. Materials and Methods

### 2.1. Materials

LA, soybean lipoxygenase type V, azelaic acid (AzA), silica G thin-layer chromatography (TLC) plates, tetramethylpentamine-2,4-dinitrophenyl hydrazine, bromocresol green, pyruvic acid, 13% BF3 in methanol, and mouse anti-human-β-actin antibody (A2228) were purchased from Sigma (St. Louis, MO, USA). Leucomethylene blue (LMB) was obtained from Alfa Aesar (Ward Hill, MA, USA). TRIzol^TM^ reagent was purchased from Invitrogen (Carlsbad, CA, USA). The goat anti-human-ApoA1 antibody was obtained from Rockland Immunochemicals (600-101-109; Gilbertsville, PA, USA). Secondary horseradish peroxidase (HRP)-conjugated rabbit anti-goat antibody was obtained from R&D Systems (BAF017; Minneapolis, MN, USA). Secondary HRP-conjugated goat anti-mouse antibody was obtained from Santa Cruz Biotechnology (SC-2039; Dallas, TX, USA). Ethyl ether containing no preservatives was purchased from Honeywell, Burdick, & Jackson (Muskegon, MI, USA). Radioactive oleic acid [^1-14^C] and LA [^1-14^C] were purchased from American Radiolabeled Chemicals (St. Louis, MO, USA).

### 2.2. Thiobarbituric Acid Reactive Substances (TBARS) Assay

The TBARS assay was used to detect malondialdehyde (MDA). TBARS reagent containing 0.67% thiobarbituric acid in 0.05N NaOH was prepared. Samples were mixed with 0.3 mL 6N HCl and 1 mL of TBARS reagent and placed in boiling water for 15 min. Samples were loaded in a 96-well plate, and absorbance was measured at 532 nm on a Benchmark Plus Microplate Absorbance Reader (Bio-Rad, Hercules, CA, USA).

### 2.3. Cell Culture

All cells were maintained at 37 °C in a humidified incubator with 5% CO_2_ and seeded in 6- and 12-well plates, depending on the experiment. Caco-2 cells, purchased from American Type Culture Collection (HTB-37; ATCC; Rockville, MD, USA), were cultured in advanced Dulbecco’s modified Eagle medium (ADMEM), supplemented with 15% fetal bovine serum (FBS), 2 mM L-glutamine, and 1% penicillin–streptomycin. Once confluent, cells were maintained in ADMEM containing 7.5% FBS. Experiments were carried out on days 3–5 (undifferentiated) and 14–21 (fully differentiated) following passaging. HepG2 cells (HB-8065; ATCC) were cultured in ADMEM supplemented with 10% FBS, 2 mM L-glutamine, and 1% penicillin–streptomycin. All cells were serum-starved 3 h prior to initiation of experimental treatments.

### 2.4. Preparation of 13-HPODE and 13-HODE

For this procedure, 13-HPODE (200 µM) and 13-HODE (200 µM) were prepared as described previously [44]. Lipid peroxides generated during the reaction were quantified via LMB assay, as previously described [50], and through measurement of conjugated dienes at 234 nm via spectrophotometer (UVIKON XL, Research Instruments International, San Diego, CA, USA). Oxidized fatty acids were extracted with BHT-free ether, dried under a stream of N_2_, and stored at −20 °C until further analysis. Radioactive [^14^C]-13-HPODE and [^14^C]-13-HODE (1000 disintegrations per minute (DPM)/nmol) were prepared in the same manner.

### 2.5. Synthesis of 9-ONA

Next, 9-ONA was prepared from oleic acid via erythro-9,10-dihydroxystearic acid (DHSA) [51,52], as the compound is not easily available commercially. Briefly, oleic acid was oxidized to DHSA using potassium permanganate and further oxidized by sodium periodate to 9-ONA. The reaction products were centrifuged at 400× *g* for 5 min at 4 °C to obtain a clear supernatant. The supernatant was dried under N_2_ and purified by TLC using a chloroform, tetrahydrofuran, and acetic acid solvent system (90:10:0.5, *v*/*v*). Product purity was assessed using gas chromatography–mass spectrometry (Clarus^®^ 560 S GC-MS, PerkinElmer, Waltham, MA, USA). Prior to cell treatment, 9-ONA was suspended in chloroform, dried under N_2_, and reconstituted in ethanol. Radioactive [^14^C]-9-ONA (1000 DPM/nmol) was synthesized from [^14^C]-oleic acid using identical methods.

### 2.6. Cell Treatment 13-HPODE and Decomposition Products

HepG2 and Caco-2 cells (poorly and fully differentiated) were seeded at an initial density of 2 × 10^5^. Caco-2 experiments were carried out on days 3–5 and 18–21 post-passaging for poorly and fully differentiated cells, respectively. Cells were initially treated with 100 µM 13-HPODE for 4 h in Hanks’ balanced salt solution (HBSS) and monitored for peroxide formation and decomposition. High-performance liquid chromatography (HPLC) and liquid chromatography–high resolution mass spectrometry (LC-HRMS) analyses were utilized to monitor the decomposition of 13-HPODE and determine resulting products, in both cell media and intracellular samples over a period of 90 min.

Following the LMB assay, HPLC and LC-HRMS confirmed that Caco-2 cells are capable of decomposing 13-HPODE to its products, and the effects of these decomposition products on cells were evaluated. Poorly and fully differentiated Caco-2 cells were treated with 13-HPODE, 9-ONA, and azelaic acid (AzA), an end product of 13-HPODE peroxidation [53], in concentrations ranging from 6.25 µM to 100 µM, for 24 h. Following the 24 h incubation, cells were harvested in TRIzol^TM^ for RNA isolation, and cell media was collected for both enzyme-linked immunosorbent assay (ELISA) analysis and paraoxonase 1 (PON1) activity assays.

### 2.7. Analyses of 13-HPODE and 13-HODE HPLC

Cell media and lysates from 100 µM 13-HPODE treatment were collected at 15 min intervals over a period of 90 min to assess 13-HPODE decomposition to 13-HODE. Analytical HPLC standards for 13-HPODE and HODE for HPLC analyses were obtained from Cayman Chemicals (10,704 and 38,600, respectively; Ann Arbor, MI, USA). Chromatographic separation of 13-HPODE and 13-HODE was achieved, as described previously [54]. Briefly, a Brownlee SPP C18 column (100 mm × 2.1 mm ID, 3 µm PS) (PerkinElmer, Waltham, MA, USA) and an isocratic mobile phase consisting of 0.3% H_3_PO_4_/acetonitrile (40:60, *v*/*v*) for 20 min with a flow rate of 1 mL/min was used to separate 13-HPODE from its decomposition product, 13-HODE. Ultraviolet (UV) absorbance was monitored at 234 nm, and peak area under the curve (AUC) was used to quantify both compounds.

### 2.8. TLC and Radioautography for Cell [^14^C]-13-HPODE Decomposition Products

At intervals following treatment of cells with [^14^C]-13-HPODE, cell media, and lysates were extracted with chloroform to determine levels of [^14^C]-13-HPODE and decomposition products, including [^14^C]-13-HODE, [^14^C]-9-ONA, and [^14^C]-AzA. Product extracts were run on a Silica G TLC plate. A chloroform:tetrahydrofuran:acetic acid (90:10:0.5, *v*/*v*) solvent system was used as the TLC mobile phase. After the separation of compounds, solvents were allowed to evaporate from the plate for 10 min. The dry TLC plate was covered with plastic wrap and exposed in the Cyclone^®^ Plus storage phosphor system (PerkinElmer, Waltham, MA, USA) for up to 48 h, with radioautography performed for each chemical spot. Each chemical spot fraction was collected and counted via scintillation counter (2450 MicroBeta^2^, PerkinElmer, Waltham, MA, USA) for the quantitative determination of [^14^C]-13-HPODE and decomposition products.

### 2.9. Extraction and LC-HRMS Analysis of 13-HPODE Decomposition Products

To confirm the formation of 13-HODE, 9-ONA, and AzA in the presence of cells, both cell media and lysates were characterized by LC-HRMS analysis (*n* = 3, in triplicates). Chloroform extraction of 13-HPODE and degradation products was performed for cell media, and the extracts were completely dried under N_2_. The resulting residues were resuspended in methanol containing 500 pmoles AzA–D14 (deuterium-labeled internal standard) to quantify AzA and other degradation products. For cell lysate lipid extraction, cells were washed twice with phosphate-buffered saline (PBS) and gently lysed in 1 mL ice-cold methanol. Lysates were further homogenized using a PowerGen700 homogenizer mixer (Fisher Scientific, Hampton, NH, USA). The resulting cell lysate lipids were extracted using 1 mL chloroform. Samples were vortexed vigorously and centrifuged at 2500 rpm for 10 min. The supernatant was collected and completely dried under N_2_. Residues were resuspended identically to those of cell media.

LC-HRMS analyses were performed on a 6520B Q-TOF (quadrupole time-of-flight) coupled with 1200 HPLC (Agilent Technologies, Santa Clara, CA, USA). Chromatographic separation of 13-HPODE and degradation products was achieved using an Agilent ZORBAX Eclipse Plus C18 (150 mm L × 4.6 mm ID, 5 µm PS) with a binary mobile phase gradient program to elute components from the column within 23 min: Eluent-A: acetonitrile and Eluent-B: deionized water, both containing 0.1% formic acid. The gradient program was as follows: 90% B: 0–3.5 min; 10% B: 3.5–12 min; 10% B, 12–19 min; 90% B: 19.1–23 min. The column was operated at 45 °C with a constant mobile phase flow rate at 0.7 mL/min. Full-scan electrospray ionization (ESI) in negative ion mode was used to ionize the molecules by applying −3.5 kV needle voltage. N_2_ was used as a drying and nebulizer gas for desolvation of solvent droplets and the values were set at 13 L/min and 55 psig, respectively; the source was operated at 320 °C, TOF fragmentor and skimmer voltages were operated at 100 V and 65 V, respectively. AzA–D14 (Cayman Chemicals, Ann Arbor, MI, USA), was used as the LC-HRMS internal standard (IS). Data were analyzed using MassHunter Quantitative Analysis B.07.00 (Agilent Technologies, Santa Clara, CA, USA).

### 2.10. Gene Expression

Total RNA from cells was isolated using TRIzol^TM^. One µg of RNA was reverse-transcribed into cDNA using the SuperScript^®^ III First-Strand Synthesis System (Invitrogen, Carlsbad, CA, USA). cDNA (50 ng) sample used to perform real-time polymerase chain reaction (RT-PCR) on an iQ^TM^5 iCycler Multicolor Real-Time PCR Detection System (Bio-Rad, Hercules, CA, USA) using SYBR Green (Invitrogen, Carlsbad, CA, USA). RT-PCR was performed for ApoA1 and PON1 using specific primers (Table S1), with glyceraldehyde-3-phosphate dehydrogenase (GAPDH) as the reference gene. Normalized fold expressions were calculated using the 2^−∆∆Ct^ method.

### 2.11. Western Blot Analysis

Total protein from cells was isolated by using radioimmunoprecipitation assay (RIPA) lysis buffer (Sigma) and stored at −20 °C until use. Protein concentration was determined using *DC* Protein Assay (Bio-Rad). Then, 8–10 µg of protein was separated on a 12% SDS–PAGE gel. The gels were transblotted onto a 0.2 µm PVDF membrane using Mini Trans-Blot Electrophoretic Transfer Cell (Bio-Rad) for 1 h at 100 V at 4 °C. Membranes were blocked with 5% nonfat milk in Tris-buffered saline (pH 7.5) with 0.1% Tween-20 (TBS-T) at room temperature for 1 h and then incubated with primary antibodies for ApoA1/PON1/ApoB (1:5000 *v*/*v* dilution) overnight at 4 °C. The blots were then incubated with secondary antibodies (1:10,000 *v*/*v* dilution) for 1 h. Finally, signals were detected using a Pierce^™^ ECL Western Blotting Substrate Kit (Thermo Technologies, Waltham, MA, USA).

### 2.12. ApoA1 and ApoB ELISAs

Cell media was collected after the indicated incubation period and centrifuged at 2000 rpm for 5 min. The supernatant was used to determine ApoA1 and ApoB levels in samples. A total of 100 µL of supernatant was analyzed for ApoA1 (3710-1HP-2; Mabtech, Cincinnati, OH, USA) and ApoB (KA1028; Abnova, Walnut, CA, USA) following the manufacturer’s protocols. Absorbance was measured at 450 nm (correction absorbance at 540 nm) using an Benchmark Plus™ Microplate Absorbance Reader (Bio-Rad). The concentration of ApoA1 and ApoB levels were expressed as secretion relative to control (untreated).

### 2.13. PON1 Activity

PON1 arylesterase activity was measured in cell media as in Jaichander et al. [55]. Briefly, 10 μL media per sample was incubated with 1 mM p-nitrophenylacetate (p-NPA) in 100 μL phosphate buffer with 2 mM CaCl_2_ and MgCl_2_ at 37 °C for 30 min in a 96-well plate. Samples were run in triplicate. p-NPA was used as the substrate for PON1 enzyme activity and the resultant product, paranitrophenol (p-NP), was measured at 410 nm on a Benchmark Plus™ Microplate Absorbance Reader (Bio-Rad).

### 2.14. Statistics

Values are presented as mean ± standard error of the mean (SE), and statistical analyses were performed using a two-tailed Student’s *t*-test or one-way analysis of variance (ANOVA), as required, at a significance level of *p* < 0.05. All experiments were completed ≥3 times. Statistics were evaluated using GraphPad Prism v5 (La Jolla, San Diego, CA, USA).

## 3. Results

### 3.1. Lipid Peroxides and Decomposition Products Detected in Dietary Cooking Oils

As the most abundant PUFA in the Western diet, LA represents a substantial potential pool for lipid peroxidation. To investigate how readily lipid peroxides and oxidative decomposition products are formed following a common deep-frying protocol, we selected three commonly used culinary oils (sesame, canola, and olive oil), varying in their PUFA percentage (45%, 20%, and 10%, respectively) to compare with butter, containing mostly saturated fats [56]. Oil samples (2 mL) were placed on a heating block at 100 °C for 8 h, and peroxide content was measured every 4 h via LMB assay. Peroxide generation increased significantly over time in both sesame oil and canola oil (Figure S1A), while olive oil and butter remained relatively consistent over the 8 h period. Sesame oil generated far fewer peroxides, compared to canola oil, despite higher levels of PUFA found in the former. This may be due to the higher antioxidant content (e.g., sesamol, vitamin E, etc.) in sesame oil. A TBARS assay was also performed to measure the presence of MDA (Figure S1B), a peroxidation product of both arachidonic acid and LA [57]. A significant increase in the amount of MDA generated over the 8 h period was observed only in canola oil.

As so little is known about the physiological effects and metabolic fate of 9-ONA, the compound was synthesized, and its properties were characterized by TLC and GC-MS analyses. The aldehyde of 9-ONA was successfully stained by DNP in a TLC system (Figure S2A), and the lipid fraction containing 9-ONA, using a solvent system of chloroform, tetrahydrofuran, and acetic acid (90:10:0.5), travels between 13-HPODE and AzA on the plate. Investigation of 9-ONA using GC-MS showed a retention time of 15.94 min (Figure S2B), and signature mass spectral peaks at 74, 87, 111, 143, and 155 (Figure S2C).

To further explore decomposition products of oxidized oils, purified 13-HPODE was left at room temperature for 48 h, and then gas chromatography was utilized to identify decomposition products of the lipid hydroperoxide. Using retention times (RTs), both 9-ONA and AzA peaks were identified in the 48 h sample (Figure S3A,C) and were compared to a gas chromatogram of freshly prepared 13-HPODE (Figure S3B–C). Peak quantification through GC-MS demonstrated significant 9-ONA and AzA peaks only in 48 h 13-HPODE samples but not in freshly prepared 13-HPODE samples (*p* < 0.01). Altogether, these results demonstrate that prolonged heating of PUFA-rich cooking oils, as occurs regularly at restaurants across the world, easily generates lipid peroxides, lipid peroxide-derived aldehydes (9-ONA), and further oxidative decomposition products (AzA).

### 3.2. Caco-2 and HepG2 Cells Efficiently Reduce Lipid Hydroperoxides to Corresponding Hydroxides

**Figure 1:**
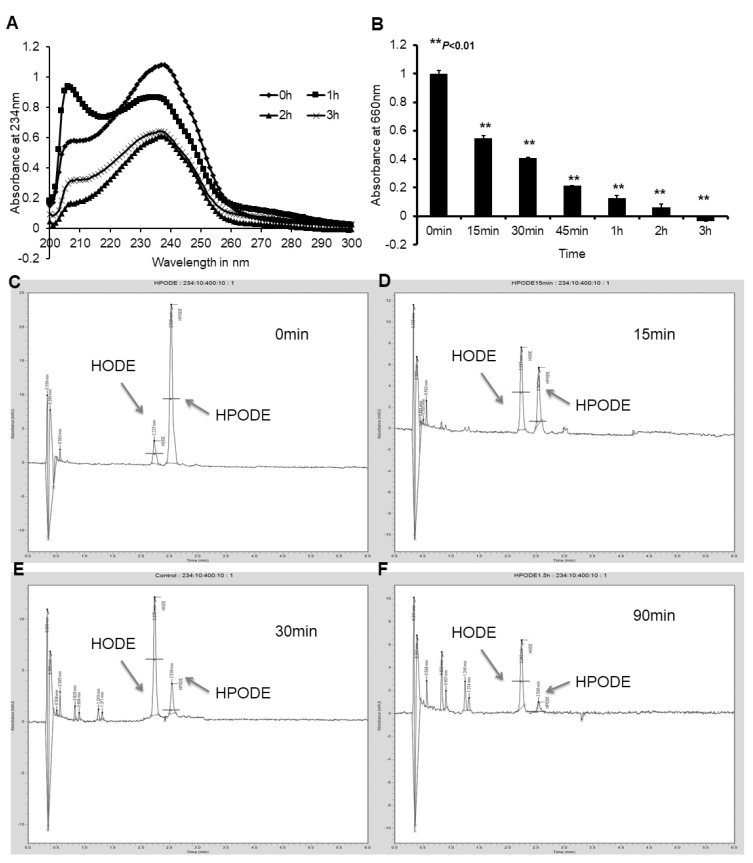
Lipid peroxides are reduced to hydroxides in the presence of fully differentiated Caco-2 cells: (**A**) 13-HPODE was incubated with differentiated Caco-2 cells, resulting in a decrease in cell media 13-HPODE dienes over 120 min; (**B**) decrease in Caco-2 cell media peroxides over time, as measured by LMB assay (** *p* < 0.01); (**C**–**F**) HPLC of Caco-2 cell media components, demonstrating a decrease in 13-HPODE and increase in 13-HODE peak AUC. Data represent 3 separate experiments, with *n* = 3/group/experiment.

In order to establish whether enterocytes alter lipid hydroperoxides, either intra- or extracellularly, freshly prepared 13-HPODE was incubated with poorly differentiated and fully differentiated Caco-2 cells, as well as with HepG2 hepatocytes. An LMB assay and conjugated diene measurements via UV spectrophotometer were performed every 15 min for 90–120 min. As shown in Figure 1A, UV spectral data demonstrated a decrease in absorbance at 234 nm, specific to conjugated dienes, indicating a loss of 13-HPODE dienes in the presence of fully differentiated Caco-2 cells. An LMB assay indicated the loss of the peroxide group from 13-HPODE in the presence of cells (Figure 1B). Comparing the results from UV spectrophotometry and the LMB assay, the peroxide group appeared to decompose more quickly than the conjugated dienes. A similar loss of peroxides was also observed in the presence of arachidonic acid 5-hydroperoxide (5-HPETE), cumene peroxide, and hydrogen peroxide (data not shown). Importantly, while these results were duplicated with both poorly differentiated Caco-2 cells and HepG2 cells, cell-free media did not accomplish the reduction of lipid hydroperoxides (Figure S4).

HPLC analysis of cell media components indicated a gradual decomposition of 13-HPODE over time. Figure 1C–F illustrates the decomposition of 13-HPODE over a period of 90 min. A significant reduction in the peak AUC for 13-HPODE was observed at 90 min (84,085.75 ± 4204 to 183.53 ± 9.2; *p* < 0.001), whereas a simultaneous increase in the 13-HODE peak AUC (1127.51 ± 56.4 to 15,721.61 ± 786; *p* < 0.001)) was detected, representing a reduction of 13-HPODE to 13-HODE.

### 3.3. Cellular Uptake of Lipid Peroxide and Corresponding Hydroxide

In order to establish whether lipid hydroperoxide reduction was occurring intracellularly or extracellularly, poorly and fully differentiated Caco-2 cells, along with HepG2, were treated with ^14^C-labeled LA, 13-HPODE, and 13-HODE to assess whether the cells were capable of taking up lipid peroxides and corresponding hydroxides. Cell lysate radioactivity was measured and standardized to cell protein concentration. Figure S5 indicates LA was more efficiently taken up by fully differentiated Caco-2 cells and HepG2 cells, compared with 13-HPODE and 13-HODE. Poorly differentiated Caco-2 cells appeared less capable of lipid uptake in general but particularly of 13-HPODE and 13-HODE. The inability of poorly differentiated Caco-2 cells to readily take up lipids is not surprising, as the brush border is essential for fatty acid absorption in the intestine [44,48].

### 3.4. TLC Radioautography of [^14^C]-13-HPODE Decomposition by Cells

**Figure 2:**
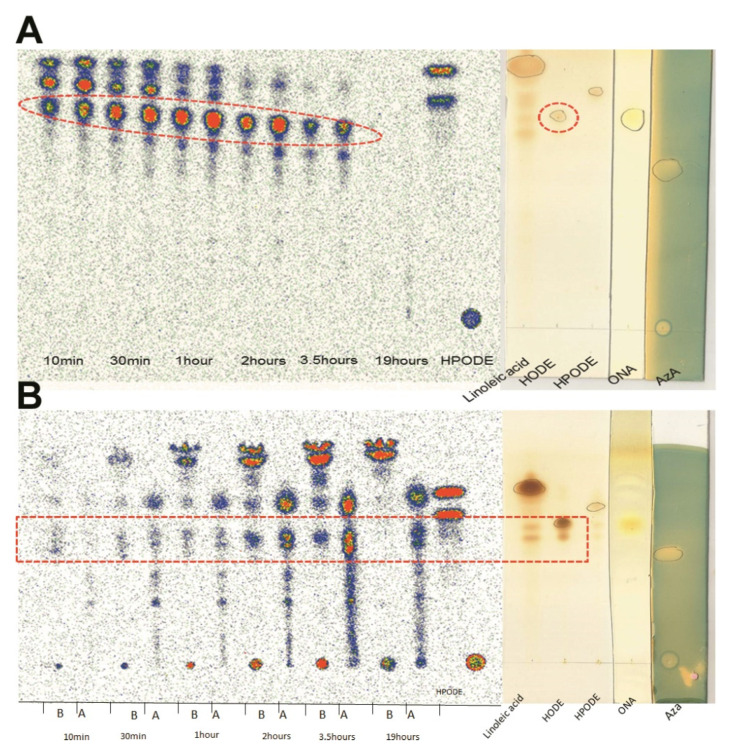
Representative TLC radioautograph of ^14^C-13-HPODE and decomposition products from fully differentiated Caco-2 cells and media: (**A**) cell media lipid extract; (**B**) intracellular lipid extracts. Dashed red line indicates 13-HODE fraction.

To examine what products resulted in intra- and extracellularly following intestinal and hepatic treatment with ^14^C-labeled LA, 13-HPODE, and 13-HODE, TLC radioautography was conducted on cell lysate and media. Radioautography data indicated differentiated Caco-2 cells decomposed ^14^C-13-HPODE into 13-HODE gradually in the media (Figure 2A; Figure S6). However, 13-HODE was gradually lost in the media and gained in the lysate, indicated the fully differentiated Caco-2 cells had taken up the compound from the media (Figure 2B). To further analyze the lipid hydroperoxide decomposition process, intracellular lipid extracts were saponified in order to hydrolyze triacylglycerols into component glycerol and fatty acids. Following saponification, fully differentiated Caco-2 cells display a denser 13-HODE fraction (Figure S7), indicating the intracellular 13-HODE derived from 13-HPODE is typically esterified as triacylglycerols.

### 3.5. LC-HRMS Analysis of 13-HPODE Decomposition Products by Caco-2 Cells

**Figure 3:**
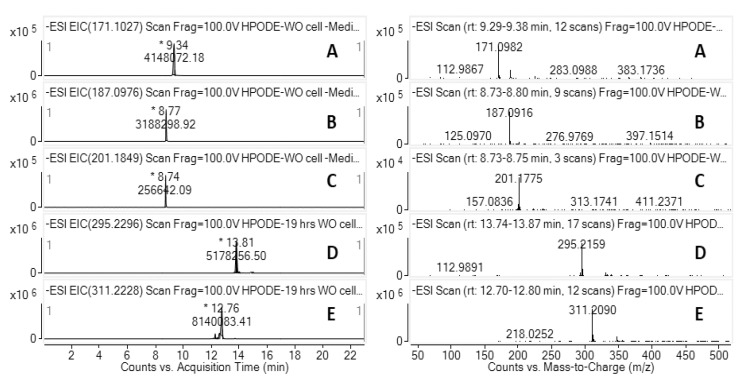
Representative overlaid LC-HRMS data from fully differentiated Caco-2 13-HPODE decomposition (*n* = 3). EIC (left panel) and ESI mass spectra (right panel) for (**A**) 9-ONA, (**B**) AzA, (**C**) AzA-D14 (IS), (**D**) 13-HODE, and (**E**) 13-HPODE.

To further characterize the decomposition products derived from cell treatment with 13-HPODE, LC-HRMS was performed on fully differentiated Caco-2 cell lysate and media (*n* = 3). In the presence of Caco-2 cells, the decomposition of 13-HPODE was observed in both cell medium and cell lysate. Chromatographic peaks were quantified by comparing to analytic standards and/or with the METLIN accurate mass database. In negative mode, deprotonated molecular ions ([M-H]^−^) at mass-to-charge ratios (*m/z*) of 311.2090, 295.2159, 187.0916, and 171.0982 were observed, corresponding to 13-HPODE, 13-HODE, AzA, and 9-ONA, respectively. Representative LC-HRMS extracted ion chromatograms (EICs) and ESI mass spectra of 13-HPODE decomposition products in a cell lysate are shown in Figure 3, with similar EIC and ESI mass spectra were seen for fully differentiated Caco-2 cell media (data not shown).

**Figure 4:**
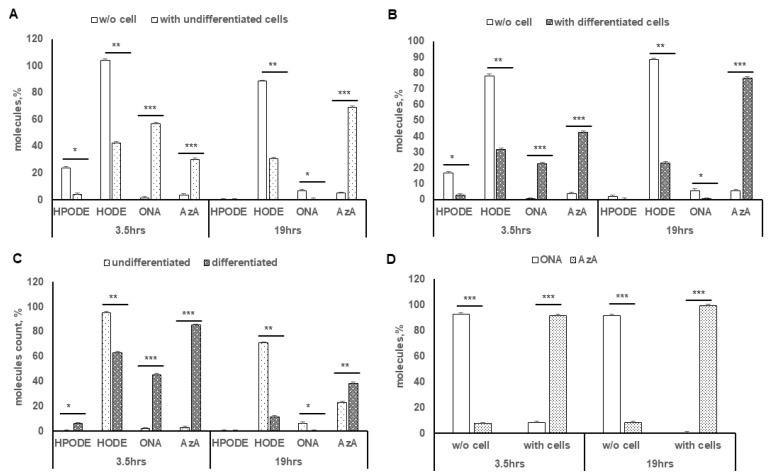
Degree of 13-HPODE and 9-ONA decomposition in media with presence and absence of Caco-2 cells at 3.5 h and 19 h post-treatment: (**A**) undifferentiated Caco-2 cells compared with cell-free media; (**B**) differentiated Caco-2 cells compared with cell-free media; (**C**) decomposition products of 13-HPODE after saponification of intracellular lipid extract from poorly and fully differentiated Caco-2 cells; (**D**) conversion of 9-ONA to AzA in presence and absence of fully differentiated Caco-2 cells. (*n* = 3; * *p* < 0.05; ** *p* < 0.01; *** *p* < 0.001).

In the presence and absence of Caco-2 cells, both poorly (Figure 4A) and fully differentiated (Figure 4B), the distribution of 13-HPODE decomposition products in cell media, identified and quantified by LC-HRMS, was measured at both 3.5 h and 19 h following 13-HPODE treatment. Undifferentiated cells generated a higher level of AzA that occurred in cell-free media, at both 3 h and 19 h, while fully differentiated Caco-2 cells mirrored this pattern to an even greater extent. In addition, the saponified intracellular lipid fraction of poorly and fully differentiated Caco-2 cells was examined (Figure 4C), with fully differentiated Caco-2 cells showing a much higher level of the intracellular decomposition products, 13-HODE and Aza, than poorly differentiated cells, especially at the 3 h mark. Finally, the conversion of 9-ONA to Aza, with and without fully differentiated Caco-2 cells (Figure 4D), demonstrated a marked increase in the ability of 9-ONA to degrade to AzA when in the presence of cells. Over 90% of treated 9-ONA was converted to AzA by 3.5 h in the presence of cells, while 98% conversion had occurred by 19 h. The concentration of intracellular AzA increased only gradually by 19 h (data not shown).

### 3.6. Oxidation of 9-ONA to AzA, and Uptake of AzA in Differentiated Caco-2 Cells

Radioactive ^14^C-9-ONA was synthesized and purified by TLC in order to trace the oxidative decomposition to AzA. In the presence of fully differentiated Caco-2 cells, a significant increase in AzA and a corresponding decrease in 9-ONA at 1 h was observed, compared with cell-free and 0 h controls (Figure S8). This indicates fully differentiated Caco-2 cells oxidize 9-ONA to AzA very efficiently in the cell medium.

To investigate the ability of Caco-2 cells to take up and utilize AzA, fully differentiated Caco-2 cells were incubated with 50 µM AzA, and metabolites of AzA were extracted from both the media and lysate for GC-MS analysis (Figure S9A). A significant increase (*p* < 0.05) in intracellular AzA was observed (Figure S9B), while levels of AzA remained detectable from 3 h to 18 h post-treatment in the cell media (Figure S9C). This result suggests the intracellular AzA concentration is tightly controlled and turnover of intracellular AzA is relatively slow. AzA stayed stable as a free fatty acid in the media until 18 h post-treatment, suggesting AzA is potentially not well absorbed by fully differentiated Caco-2 cells.

### 3.7. Intestinal and Hepatic 13-HPODE-, 9-ONA-, and AzA-Induced ApoA1 Secretion

**Figure 5:**
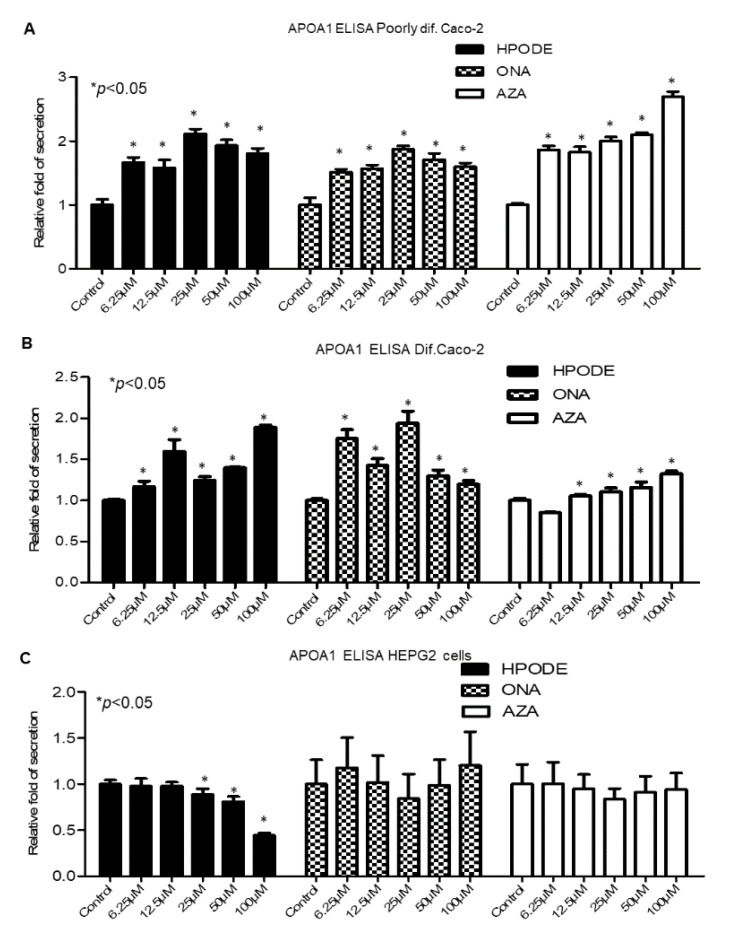
Caco-2 and HepG2 secretion of ApoA1 following incubation with 13-HPODE, 9-ONA and AzA for 24 h: (**A**) poorly differentiated Caco-2 cells; (**B**) fully differentiated Caco-2 cells; (**C**) HepG2 cells (*n* = 9/group; * *p* < 0.05).

In order to determine any effect of dietary oxidized lipids on ApoA1 secretion, poorly and fully differentiated Caco-2 cells, as well as HepG2, were treated with 13-HPODE (0–100 µM) for 6 or 20 h, and gene and protein expression for ApoA1 was evaluated. ApoA1 gene expression in the intestine did not show consistent trends (Figure S10), while expression in HepG2 cells was completely unaltered (data not shown). To evaluate a potential protein response to treatment, an ELISA for ApoA1 was performed using cell media 24 h post-treatment. In poorly differentiated Caco-2 cells, a significant (*p* < 0.05) increase in ApoA1 levels was observed with 13-HPODE, 9-ONA, and AzA treatment (Figure 5A), while similar increases were observed in fully differentiated Caco-2 cells (Figure 5B). Interestingly, secretion of ApoA1 protein in response to 13-HPODE in HepG2 cells decreased significantly (Figure 5C), with no effect seen from 9-ONA or AzA, suggesting protein expression of ApoA1 in Caco-2 and HepG2 cells are differentially regulated. Western blots for fully differentiated Caco-2 cell lysates were performed, with no significant observed inductions of intracellular ApoA1 following 13-HPODE, 9-ONA, or AzA treatments (data not shown).

### 3.8. Intestinal and Hepatic 13-HPODE-, 9-ONA-, and Aza-Induced PON1 Activity

**Figure 6:**
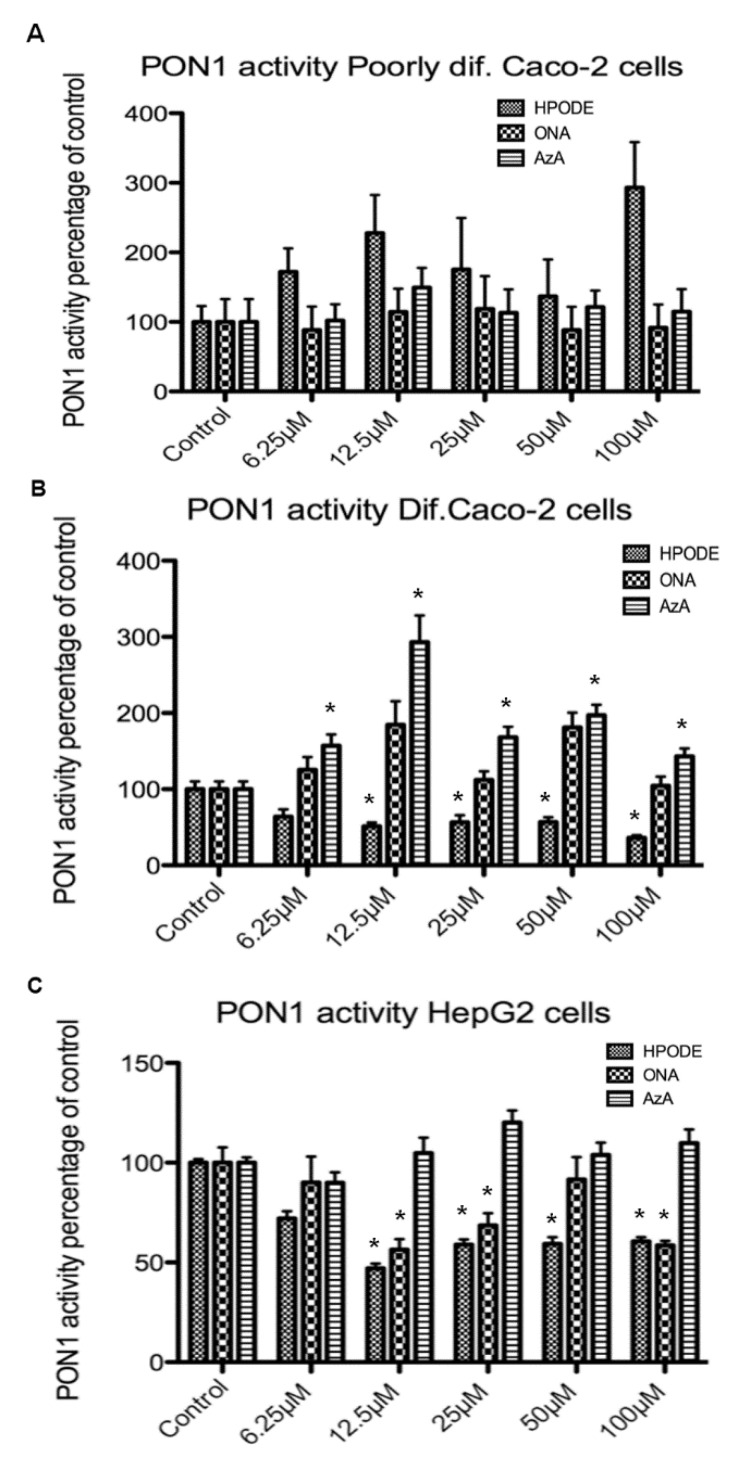
PON1 activity following treatment with 13-HPODE, 9-ONA and AzA: (**A**) poorly differentiated Caco-2 cells; (**B**) fully differentiated Caco-2 cells; (**C**) HepG2 cells (*n* = 9/group, * *p* < 0.05).

As an antioxidant enzyme closely associated with HDL and capable of hydrolyzing a variety of oxidative compounds, PON1 is considered antiatherogenic [58]. To investigate any potential influence on PON1 activity following lipid peroxide treatment, poorly and fully differentiated Caco-2 cells, as well as HepG2, were treated with 13-HPODE, 9-ONA, and AzA, and the cell media was subjected to a PON1 activity assay. No significant PON1 activity changes following treatment were observed in the media of poorly differentiated Caco-2 cells, potentially due to low total enzyme activity in the medium (Figure 6A). A significant (*p* < 0.05) decrease in PON1 activity with an increasing concentration of 13-HPODE was seen with fully differentiated Caco-2 cells. While 9-ONA treatment did not influence PON1 activity in fully differentiated Caco-2 cells, treatment with AzA significantly (*p* < 0.05) increased PON1 activity in the media of these cells (Figure 6B). In HepG2 cell media, decreased PON1 activity was observed following 13-HPODE and 9-ONA treatment, whereas no change was observed with treatment by AzA (Figure 6C). While 13-HPODE did significantly suppress PON1 activity, at the level of the gene, this oxidized lipid did not inhibit PON1 expression in any of the three cell types (data not shown). Reduced PON1 activity is associated with dysfunctional HDL, and the presence of 13-HPODE may provide a source and context for loss of HDL functionality. Moreover, 9-ONA treatments had inconsistent effects among cell types, with a trend toward increased and decreased PON1 activity in differentiated Caco-2 cells and HepG2, respectively. AzA significantly enhanced PON1 activity in differentiated Caco-2 cell media but not in the media of poorly differentiated Caco-2 or HepG2 cells.

### 3.9. Suppression of ApoB48 Secretion by AzA in Differentiated Caco-2 Cells

To further interrogate the potential lipoprotein effects of dietary peroxidized lipid interaction with intestinal and hepatic cells, the secretion of ApoB48 and ApoB100 following treatment with 13-HPODE, 9-ONA, and AzA was investigated. Using an antibody that binds to the N-terminal of the ApoB protein, an ELISA was performed for poorly and fully differentiated Caco-2 cell media, as well as for HepG2. Poorly differentiated Caco-2 cells secreted very little ApoB48 into the medium, resulting in very low readings (data not shown). In fully differentiated Caco-2 cells and HepG2 cells, 100 μM 13-HPODE inhibits ApoB secretion after 24 h (Figure S11). In fully differentiated Caco-2 cells, a concentration-dependent decrease in ApoB secretion was observed with 13-HPODE treatment, a trend not mirrored in HepG2 cells, suggesting intestinal cells may be more sensitive to externally supplied 13-HPODE. ApoB secretion following 100 μM 9-ONA treatment was significantly decreased (*p* < 0.05) in fully differentiated Caco-2 cells (Figure S11A). A significant decrease (*p* < 0.05) in ApoB secretion in fully differentiated Caco-2 cells, at both 50 and 100 μM AzA (Figure S11A), was also observed. ApoB secretion following 100 μM 13-HPODE treatment was significantly suppressed in HepG2 cells (Figure S11B). However, treatment with either 9-ONA or AzA did not influence ApoB secretion by HepG2.

## 4. Discussion

Earlier studies have suggested dietary peroxidized lipids may be atherogenic [59,60]. As we have, in part, demonstrated, the large quantities of PUFA-containing cooking oils common in Western diets can produce peroxidized lipids when subjected to extended periods of heating, a potentially important health risk given the popularity of fried foods. The small intestine, as the site of dietary lipid absorption, plays a major role in the formation and transportation of lipids, primarily chylomicrons, to tissues throughout the body [61].

As the only enterocyte model capable of differentiation to the point of physiologically relevant lipoprotein processing [62], Caco-2 cells are a popular tissue culture model for intestinal lipoprotein studies [63,64]. This cell model has established important physiology relating to fatty acid post-transcriptional regulation of ApoB production, lipoprotein production, and transintestinal cholesterol efflux (TICE), a pathway by which intestinal cholesterol uptake is limited, ApoA1 and HDL levels are increased, and cholesterol efflux into the intestinal lumen is increased [65]. While the intestinal metabolism of lipids has been considered, that of peroxidized lipids has not. A few studies have suggested dietary peroxidized lipids can be absorbed by the small intestine, thereby contributing to the formation of circulating, potentially atherogenic, oxidized lipoproteins (e.g., [59]). In contrast, studies from our laboratory have shown induction of ApoA1 by 4d and 14d Caco-2 cells when incubated with oxidized LA (13-HPODE) [44]. While intestinal absorption of 13-HPODE would presumably be atherogenic, decomposition of this oxidized lipid to aldehydes and dicarboxylic acids could be beneficial in upregulating the antiatherogenic ApoA1. Thus, our current study attempted to determine the fate of 13-HPODE in both intestinal and liver cells, with a secondary goal of determining an effect on ApoA1 expression as a result of lipid peroxide decomposition products.

Based on our results, we pose the question of whether dietary oxidized fatty acids are friends or foes, either individually or via their metabolic products. Further, we question whether dietary oxidized fatty acids may create additional, externally derived oxidative stress and exacerbate the atherogenic process. On the other hand, we would like to point out that the paradoxical idea that dietary oxidized lipids or their decomposition products act as peroxisome proliferator-activated receptor (PPAR) α agonists yet appear to enhance the production of ApoA1/PON1 (the major components of HDL) by intestinal cells.

By studying the metabolic fate of 13-HPODE, we concluded that, in the presence of Caco-2 cells, 13-HPODE is reduced to 13-HODE efficiently. Various factors, such as thiol-containing peptides, amino acids, and antioxidants may contribute to the reduction of 13-HPODE [66]. In the context of dietary peroxide lipids, which are hydrolyzed by a variety of lipases (mostly pancreatic lipase) to produce free fatty acid peroxides in the gastrointestinal tract, dietary free fatty acid peroxides are present in a mixture in chime, along with amino acids, peptides, and antioxidants from the diet. Within 3–4 h post-digestion, fatty acid peroxides would be expected to have been largely reduced by dietary components and via interaction with intestinal cells. Yuan et al. [67] reported 9-HODE and 13-HODE are the two major metabolites from oxidized LA in rat plasma using MS-based studies. However, Kanazawa and Ashida [68] showed 13-HPODE was released from TG in the stomach and decomposed to aldehydes before reaching the small intestine. We found 13-HPODE is unstable at room temperature and autodecomposes into aldehydes and, subsequently, dicarboxylic acids. Significant amounts of aldehydes were detected by GC-MS derived from 13-HPODE samples stored at room temperature for 48 h. In general, food remains in the stomach for approximately 2 h, leading to the expectation that decomposition or aldehyde generation would not be significant in the intestine, however significant the amount of ingested lipid peroxides. Our findings suggested dietary peroxides may not be a significant source of lipid peroxides in the circulation, as they can be reduced to corresponding hydroxides during the digestion process. Some in vivo studies demonstrated oxidative stress was induced by consuming heated oils, potentially due to the secondary effects of lipid peroxide-derived products rather than the original compounds (e.g., [69]). We have demonstrated here, however, that 13-HPODE decreases PON1 activity, potentially preventing a source of significant antioxidant activity.

This is the first study to demonstrate the metabolic fate of 9-ONA, the decomposition product from the carboxylic end of, likely, the most abundant lipid peroxide in nature, linoleic peroxide (13-HPODE). LC-HRMS and ^14^C-labeled radioautography techniques were used to evaluate the oxidation of 9-ONA to AzA in the presence of Caco-2 cells. The accumulation of AzA in the extracellular medium suggests plasma membrane-bound aldehyde oxidizing enzymes may play a role in this reaction. Many enzymes, such as the gene superfamilies of aldehyde dehydrogenases and oxidases [70], may be involved in the oxidation of fatty aldehydes.

AzA is a potentially antiatherosclerotic compound [53]. This is the first study to demonstrate AzA is poorly absorbed by intestinal cells and suggests that AzA has an efficient modulatory property of lipoprotein secretion by intestinal cells. AzA effectively enhanced ApoA1 secretion and PON1 activity, the main components of HDL, while suppressing ApoB secretion in differentiated Caco-2 cells. However, AzA did not modulate lipoprotein secretion in hepatocytes.

AzA remained detectable in differentiated Caco-2 cell medium for up to 18 h, while LC-HRMS confirmed less than 5% of treated AzA was taken up by cells. Grego et al. [71] found intravenous (*i.v*) infusion of AzA resulted in more than a 50% loss through the urine and suggested AzA was not suitable as an energy substrate in parenteral nutrition. Passi et al. [72] also suggested AzA serum concentrations and urinary excretion are high when the compound was infused intravenously or intraarterially, as compared to oral administration. AzA has been ascribed several beneficial properties, including anti-inflammatory, antimicrobial, antitumoral, and anti-keratinizing effects [53,73]. AzA has also been used in various formulations to treat rosacea, acne, and melisma [74]. Litvinov et al. [53] suggested ingestion of AzA could slow the progress of atherosclerosis; however, the mechanism by which AzA reduces atherosclerosis was unclear. As demonstrated here, AzA has minimal effect on hepatic cells, and poor absorption by intestinal cells, potentially accounting for the beneficial effects of AzA being restricted to the intestine. Thus, the intestine may be a novel target for antiatherosclerotic drugs.

## 5. Conclusions

This study sheds light on the intestine as a novel organ through which to target potentially atherosclerotic mechanisms. The regulation of intestinal lipoprotein secretion appears to differ from that of the liver. Our findings suggest the existence of intestine-specific modulators at the post-transcriptional level that may modify lipoprotein production. However, this process is not yet completely understood, opening new avenues for further investigation. Considering derivatives of AzA have been widely used in the treatment of inflammatory bowel disease (IBD), which enhances the progression of atherosclerosis [75], these results may also interest the gastrointestinal research community.

## Data Availability

Data are contained within the article and supplementary material.

## Supplementary Materials

The Supplementary Materials are available online at https://www.mdpi.com/article/10.3390/antiox10081258/s1.

## References

[1] Sacks F.M., Lichtenstein A.H., Wu J.H.Y., Appel L.J., Creager M.A., Kris-Etherton P.M., Miller M., Rimm E.B., Rudel L.L., Robinson J.G. (2017). Dietary Fats and Cardiovascular Disease: A Presidential Advisory From the American Heart Association. Circulation.

[2] Kuller L.H. (2006). Nutrition, lipids, and cardiovascular disease. Nutr. Rev..

[3] Chahoud G., Aude Y.W., Mehta J.L. (2004). Dietary recommendations in the prevention and treatment of coronary heart disease: Do we have the ideal diet yet?. Am. J. Cardiol..

[4] Lichtenstein A.H. (2003). Dietary fat and cardiovascular disease risk: Quantity or quality?. J. Women’s Health.

[5] Lemieux H., Bulteau A.L., Friguet B., Tardif J.C., Blier P.U. (2011). Dietary fatty acids and oxidative stress in the heart mitochondria. Mitochondrion.

[6] Behniwal P.K., Soni G.L., Vadhera S., Singh R. (1993). In vitro absorption of nutrients from small intestine of rats fed peroxidized oil. Indian J. Exp. Biol..

[7] Grootveld M., Percival B.C., Grootveld K.L. (2018). Chronic non-communicable disease risks presented by lipid oxidation products in fried foods. Hepatobiliary Surg. Nutr..

[8] Glavind J., Hartmann S. (1951). The occurrence of peroxidized lipids in atheromatous human aortas. Experientia.

[9] Desai I.D., Tappel A.L. (1963). Damage to proteins by peroxidized lipids. J. Lipid Res..

[10] Nielsen H. (1978). Reaction between peroxidized phospholipid and protein: I. Covalent binding of peroxidized cardiolipin to albumin. Lipids.

[11] Nielsen H. (1979). Reaction between peroxidized phospholipid and protein: II. Molecular weight and phosphorus content of albumin after reaction with peroxidized cardiolipin. Lipids.

[12] Barsacchi R., Camici P., Bottigli U., Salvadori P.A., Pelosi G., Maiorino M., Ursini F. (1983). Correlation between hydroperoxide-induced chemiluminescence of the heart and its function. Biochim. Biophys. Acta.

[13] Gulati J., Gupta P.P., Soni G.L., Vadhera S., Singh R. (1992). Effects of peroxidized oil on the development of experimental atherosclerosis in rabbits. Indian Heart J..

[14] Mickel H.S., Horbar J. (1974). The effect of peroxidized arachidonic acid upon human platelet aggregation. Lipids.

[15] Mukai F.H., Goldstein B.D. (1976). Mutagenicity of malonaldehyde, a decomposition product of peroxidized polyunsaturated fatty acids. Science.

[16] Hegstad A.C., Strand H., Ytrehus K. (1994). Phospholipid peroxidation after 60 min of global ischaemia and 10 min of reperfusion. A study in the isolated rat heart. J. Mol. Cell. Cardiol..

[17] Ytrehus K., Aspang E.M. (1994). Phospholipid peroxidation in isolated perfused rat hearts subjected to hypothermia followed by rewarming: Inverse relation to loss of function. Cryobiology.

[18] Goheen S.C., O’Rourke L., Larkin E.C. (1986). Ozone and the peroxidation of polyunsaturated fatty acids in vivo. Environ. Res..

[19] Zalejska-Fiolka J., Wielkoszyński T., Kasperczyk S., Kasperczyk A., Birkner E. (2012). Effects of oxidized cooking oil and α-lipoic acid on blood antioxidants: Enzyme activities and lipid peroxidation in rats fed a high-fat diet. Biol. Trace Elem. Res..

[20] Yin H., Porter N.A. (2005). New insights regarding the autoxidation of polyunsaturated fatty acids. Antioxid. Redox Signal..

[21] Fritsche K.L. (2014). Linoleic acid, vegetable oils & inflammation. MO Med..

[22] Whelan J., Fritsche K. (2013). Linoleic acid. Adv. Nutr..

[23] Raghavamenon A., Garelnabi M., Babu S., Aldrich A., Litvnov D., Parthasarathy S. (2009). a-Tocopherol Is Ineffective in Preventing the Decomposition of Preformed Lipid Peroxides and May Promote the Accumulation of Toxic Aldehydes: A Potential Explanation for the Failure of Antioxidants to Affect Human Atherosclerosis. Antioxid. Redox Signal..

[24] Spickett C.M. (2013). The lipid peroxidation product 4-hydroxy-2-nonenal: Advances in chemistry and analysis. Redox Biol..

[25] Poli G., Biasi F., Leonarduzzi G. (2008). 4-Hydroxynonenal-protein adducts: A reliable biomarker of lipid oxidation in liver diseases. Mol. Asp. Med..

[26] Khan-Merchant N., Penumetcha M., Meilhac O., Parthasarathy S. (2002). Oxidized fatty acids promote atherosclerosis only in the presence of dietary cholesterol in low-density lipoprotein receptor knockout mice. J. Nutr..

[27] Feig J.E., Feig J.L., Dangas G.D. (2016). The role of HDL in plaque stabilization and regression: Basic mechanisms and clinical implications. Coron. Artery Dis..

[28] Pownall H.J., Ehnholm C. (2006). The unique role of apolipoprotein A-I in HDL remodeling and metabolism. Curr. Opin. Lipidol..

[29] Brouillette C.G., Anantharamaiah G.M., Engler J.A., Borhani D.W. (2001). Structural models of human apolipoprotein A-I: A critical analysis and review. Biochim. Biophys. Acta.

[30] Colvin P.L., Parks J.S. (1999). Metabolism of high density lipoprotein subfractions. Curr. Opin. Lipidol..

[31] Eggerman T.L., Hoeg J.M., Meng M.S., Tombragel A., Bojanovski D., Brewer H.B. (1991). Differential tissue-specific expression of human apoA-I and apoA-II. J. Lipid Res..

[32] Danielsen E.M., Hansen G.H., Rasmussen K., Niels-Christiansen L.L., Frenzel F. (2012). Apolipoprotein A-1 (apoA-1) deposition in, and release from, the enterocyte brush border: A possible role in transintestinal cholesterol efflux (TICE)?. Biochim. Biophys. Acta.

[33] Rader D.J. (2003). High-density lipoproteins as an emerging therapeutic target for atherosclerosis. JAMA J. Am. Med. Assoc..

[34] Franceschini G. (2001). Epidemiologic evidence for high-density lipoprotein cholesterol as a risk factor for coronary artery disease. Am. J. Cardiol..

[35] Maron D.J. (2000). The epidemiology of low levels of high-density lipoprotein cholesterol in patients with and without coronary artery disease. Am. J. Cardiol..

[36] Libby P., Aikawa M., Schonbeck U. (2000). Cholesterol and atherosclerosis. Biochim. Biophys. Acta.

[37] Navab M., Anantharamaiah G.M., Reddy S.T., Van Lenten B.J., Ansell B.J., Fogelman A.M. (2006). Mechanisms of disease: Proatherogenic HDL—An evolving field. Nat. Clin. Practice. Endocrinol. Metab..

[38] Duffy D., Rader D.J. (2006). Emerging therapies targeting high-density lipoprotein metabolism and reverse cholesterol transport. Circulation.

[39] Curtiss L.K., Valenta D.T., Hime N.J., Rye K.A. (2006). What is so special about apolipoprotein AI in reverse cholesterol transport?. Arterioscler. Thromb. Vasc. Biol..

[40] Trigatti B., Rigotti A., Krieger M. (2000). The role of the high-density lipoprotein receptor SR-BI in cholesterol metabolism. Curr. Opin. Lipidol..

[41] Parthasarathy S., Barnett J., Fong L.G. (1990). High-density lipoprotein inhibits the oxidative modification of low-density lipoprotein. Biochim. Biophys. Acta.

[42] Navab M., Ananthramaiah G.M., Reddy S.T., Van Lenten B.J., Ansell B.J., Fonarow G.C., Vahabzadeh K., Hama S., Hough G., Kamranpour N. (2004). The oxidation hypothesis of atherogenesis: The role of oxidized phospholipids and HDL. J. Lipid Res..

[43] Navab M., Hama S.Y., Cooke C.J., Anantharamaiah G.M., Chaddha M., Jin L., Subbanagounder G., Faull K.F., Reddy S.T., Miller N.E. (2000). Normal high density lipoprotein inhibits three steps in the formation of mildly oxidized low density lipoprotein: Step 1. J. Lipid Res..

[44] Rong R., Ramachandran S., Penumetcha M., Khan N., Parthasarathy S. (2002). Dietary oxidized fatty acids may enhance intestinal apolipoprotein A-I production. J. Lipid Res..

[45] Cohn J.S., Marcoux C., Davignon J. (1999). Detection, quantification, and characterization of potentially atherogenic triglyceride-rich remnant lipoproteins. Arterioscler. Thromb. Vasc. Biol..

[46] Olofsson S.O., Borèn J. (2005). Apolipoprotein B: A clinically important apolipoprotein which assembles atherogenic lipoproteins and promotes the development of atherosclerosis. J. Intern. Med..

[47] Staprãns I., Rapp J.H., Pan X.M., Kim K.Y., Feingold K.R. (1994). Oxidized lipids in the diet are a source of oxidized lipid in chylomicrons of human serum. Arter. Thromb..

[48] Penumetcha M., Khan N., Parthasarathy S. (2000). Dietary oxidized fatty acids: An atherogenic risk?. J. Lipid Res..

[49] Porter N.A. (2013). A perspective on free radical autoxidation: The physical organic chemistry of polyunsaturated fatty acid and sterol peroxidation. J. Org. Chem..

[50] Ohishi N., Ohkawa H., Miike A., Tatano T., Yagi K. (1985). A new assay method for lipid peroxides using a methylene blue derivative. Biochem. Int..

[51] Kaneko T., Kaji K., Matsuo M. (1988). Cytotoxicities of a linoleic acid hydroperoxide and its related aliphatic aldehydes toward cultured human umbilical vein endothelial cells. Chem. Biol. Interact..

[52] Lopez M.A., Vicente J., Kulasekaran S., Vellosillo T., Martinez M., Irigoyen M.L., Cascon T., Bannenberg G., Hamberg M., Castresana C. (2011). Antagonistic role of 9-lipoxygenase-derived oxylipins and ethylene in the control of oxidative stress, lipid peroxidation and plant defence. Plant J. Cell Mol. Biol..

[53] Litvinov D., Selvarajan K., Garelnabi M., Brophy L., Parthasarathy S. (2010). Anti-atherosclerotic actions of azelaic acid, an end product of linoleic acid peroxidation, in mice. Atherosclerosis.

[54] Muellner M.K., Schreier S.M., Laggner H., Hermann M., Esterbauer H., Exner M., Gmeiner B.M., Kapiotis S. (2009). Hydrogen sulfide destroys lipid hydroperoxides in oxidized LDL. Biochem. J..

[55] Jaichander P., Selvarajan K., Garelnabi M., Parthasarathy S. (2008). Induction of paraoxonase 1 and apolipoprotein A-I gene expression by aspirin. J. Lipid Res..

[56] Orsavova J., Misurcova L., Ambrozova J.V., Vicha R., Mlcek J. (2015). Fatty Acids Composition of Vegetable Oils and Its Contribution to Dietary Energy Intake and Dependence of Cardiovascular Mortality on Dietary Intake of Fatty Acids. Int. J. Mol. Sci..

[57] Yamauchi Y., Furutera A., Seki K., Toyoda Y., Tanaka K., Sugimoto Y. (2008). Malondialdehyde generated from peroxidized linolenic acid causes protein modification in heat-stressed plants. Plant Physiol. Biochem..

[58] Shokri Y., Variji A., Nosrati M., Khonakdar-Tarsi A., Kianmehr A., Kashi Z., Bahar A., Bagheri A., Mahrooz A. (2020). Importance of paraoxonase 1 (PON1) as an antioxidant and antiatherogenic enzyme in the cardiovascular complications of type 2 diabetes: Genotypic and phenotypic evaluation. Diabetes Res. Clin. Pract..

[59] Staprans I., Hardman D.A., Pan X.M., Feingold K.R. (1999). Effect of oxidized lipids in the diet on oxidized lipid levels in postprandial serum chylomicrons of diabetic patients. Diabetes Care.

[60] Staprans I., Pan X.M., Rapp J.H., Feingold K.R. (2005). The role of dietary oxidized cholesterol and oxidized fatty acids in the development of atherosclerosis. Mol. Nutr. Food Res..

[61] Iqbal J., Hussain M.M. (2009). Intestinal lipid absorption. Am. J. Physiol. Endocrinol. Metab..

[62] Levy E., Mehran M., Seidman E. (1995). Caco-2 cells as a model for intestinal lipoprotein synthesis and secretion. FASEB J..

[63] Hiebl V., Schachner D., Ladurner A., Heiss E.H., Stangl H., Dirsch V.M. (2020). Caco-2 Cells for Measuring Intestinal Cholesterol Transport—Possibilities and Limitations. Biol. Proced. Online.

[64] Mehran M., Levy E., Bendayan M., Seidman E. (1997). Lipid, apolipoprotein, and lipoprotein synthesis and secretion during cellular differentiation in Caco-2 cells. In Vitro Cell Dev. Biol. Anim..

[65] Grefhorst A., Verkade H.J., Groen A.K. (2019). The TICE Pathway: Mechanisms and Lipid-Lowering Therapies. Methodist Debakey Cardiovasc. J..

[66] Aw T.Y., Williams M.W. (1992). Intestinal absorption and lymphatic transport of peroxidized lipids in rats: Effect of exogenous GSH. Am. J. Physiol..

[67] Yuan Z.X., Rapoport S.I., Soldin S.J., Remaley A.T., Taha A.Y., Kellom M., Gu J., Sampson M., Ramsden C.E. (2013). Identification and profiling of targeted oxidized linoleic acid metabolites in rat plasma by quadrupole time-of-flight mass spectrometry. Biomed. Chromatogr. BMC.

[68] Kanazawa K., Ashida H. (1998). Dietary hydroperoxides of linoleic acid decompose to aldehydes in stomach before being absorbed into the body. Biochim. Biophys. Acta.

[69] Zarei M., Uppin V., Acharya P., Talahalli R. (2021). Ginger and turmeric lipid-solubles attenuate heated oil-induced oxidative stress in the brain via the upregulation of NRF2 and improve cognitive function in rats. Metab. Brain Dis..

[70] Jackson B., Brocker C., Thompson D.C., Black W., Vasiliou K., Nebert D.W., Vasiliou V. (2011). Update on the aldehyde dehydrogenase gene (ALDH) superfamily. Hum. Genom..

[71] Grego A.V., Mingrone G. (1995). Dicarboxylic acids, an alternate fuel substrate in parenteral nutrition: An update. Clin. Nutr..

[72] Passi S., Picardo M., Mingrone G., Breathnach A.S., Nazzaro-Porro M. (1989). Azelaic acid--biochemistry and metabolism. Acta Derm. Venereol. Suppl..

[73] Fitton A., Goa K.L. (1991). Azelaic acid. A review of its pharmacological properties and therapeutic efficacy in acne and hyperpigmentary skin disorders. Drugs.

[74] Sieber M.A., Hegel J.K. (2014). Azelaic acid: Properties and mode of action. Ski. Pharmacol. Physiol..

[75] Schicho R., Marsche G., Storr M. (2015). Cardiovascular complications in inflammatory bowel disease. Curr. Drug Targets.

