# Supplementary material for: Intestinal and Hepatic Uptake of Dietary Peroxidized Lipids and Their Decomposition Products, and Their Subsequent Effects on Apolipoprotein A1 and Paraoxonase1
Source: Antioxidants (Basel). 2021 Aug 6;10(8):1258. doi: 10.3390/antiox10081258 (PMC8389297; doi:10.3390/antiox10081258)
Supplement: Supplementary file 1 [file antioxidants-10-01258-s001.zip › antioxidants-1280072-supplementary.pdf]

Supplementary Tables and Figures:

Table S1. RT-PCR Primer Sequences

Primer	Sequence (5' to 3')
ApoA1	Forward: TGGGATCGAGTGAAGGACCT<br>Reverse: CTCCTCCTGCCACTTCTTCTG
PON1	Forward: CTCACTGAGGCGGTCATGTT<br>Reverse: TAGGCTTTGCTGTCCTGAGC
GAPDH	Forward: AGTCAACGGATTTGGTCGTA<br>Reverse: GGAACATGTAAACCATGTAGTTGAG

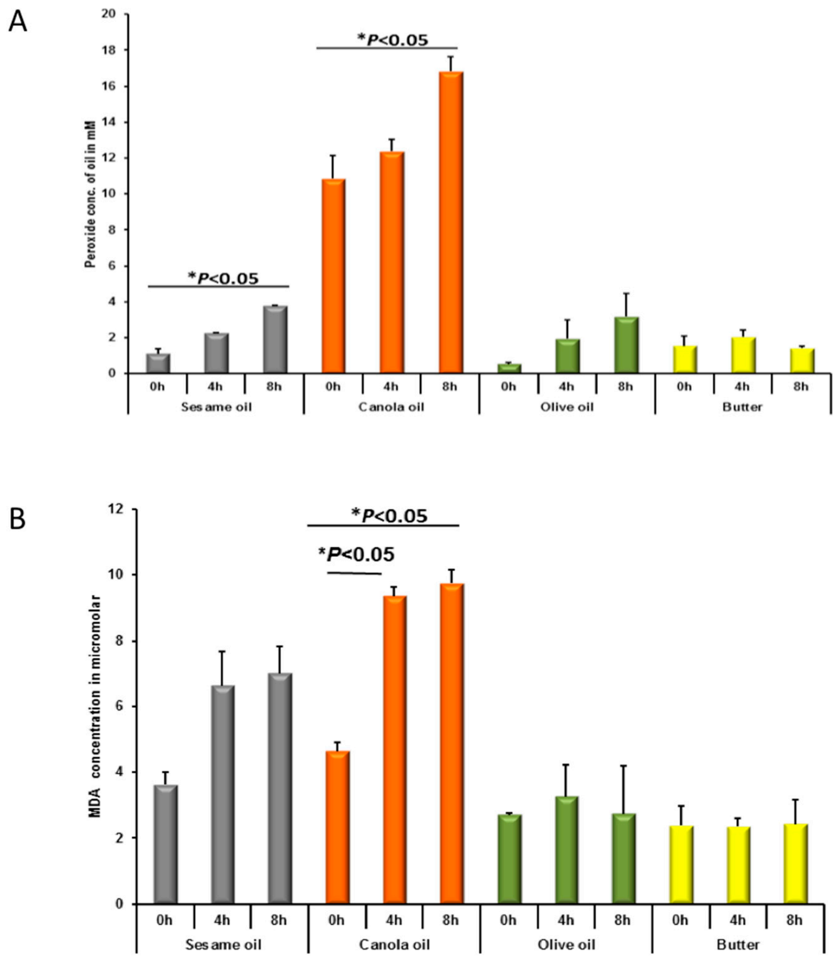

Figure S1. **Peroxide and MDA content in cooking oils and butter following heating.** Peroxide content **(A)** was determined by LMB assay (n = 3/oil) and MDA content **(B)** was determined by TBARS (n = 3/oil). Peroxide content increased significantly during the 8hr cooking period, in both canola and sesame oil ($p < 0.05$). MDA content also increased significantly during the cooking period in canola oil ($p < 0.05$).

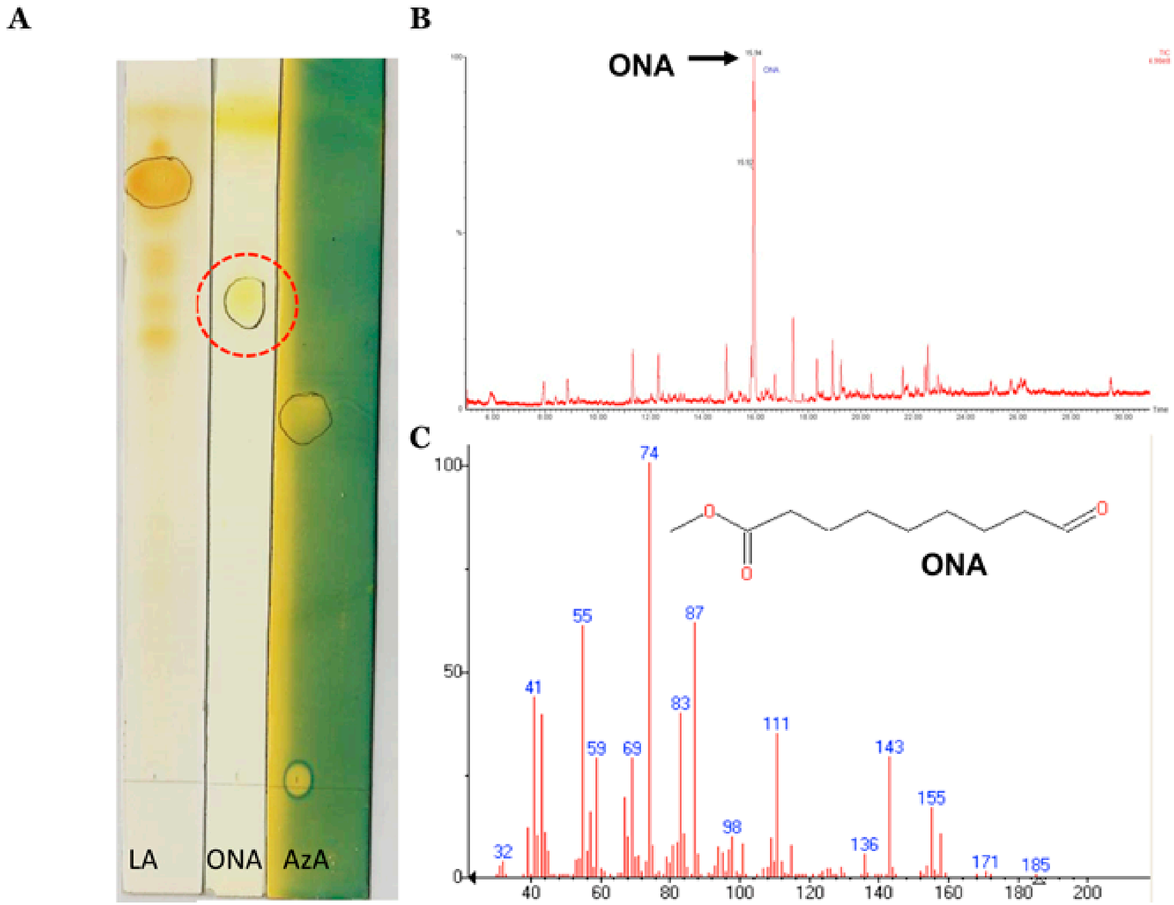

SFigure S2. Characterization of 9-ONA synthesized in the laboratory. (A) TLC of 9-ONA (dashed circle) in solvent system of chloroform, tetrahydrofuran, and acetic acid (90:10:0.5). **(B)** Gas chromatogram of synthesized 9-ONA (RT of 15.94min). **(C)** Mass spectrum of 9-ONA.

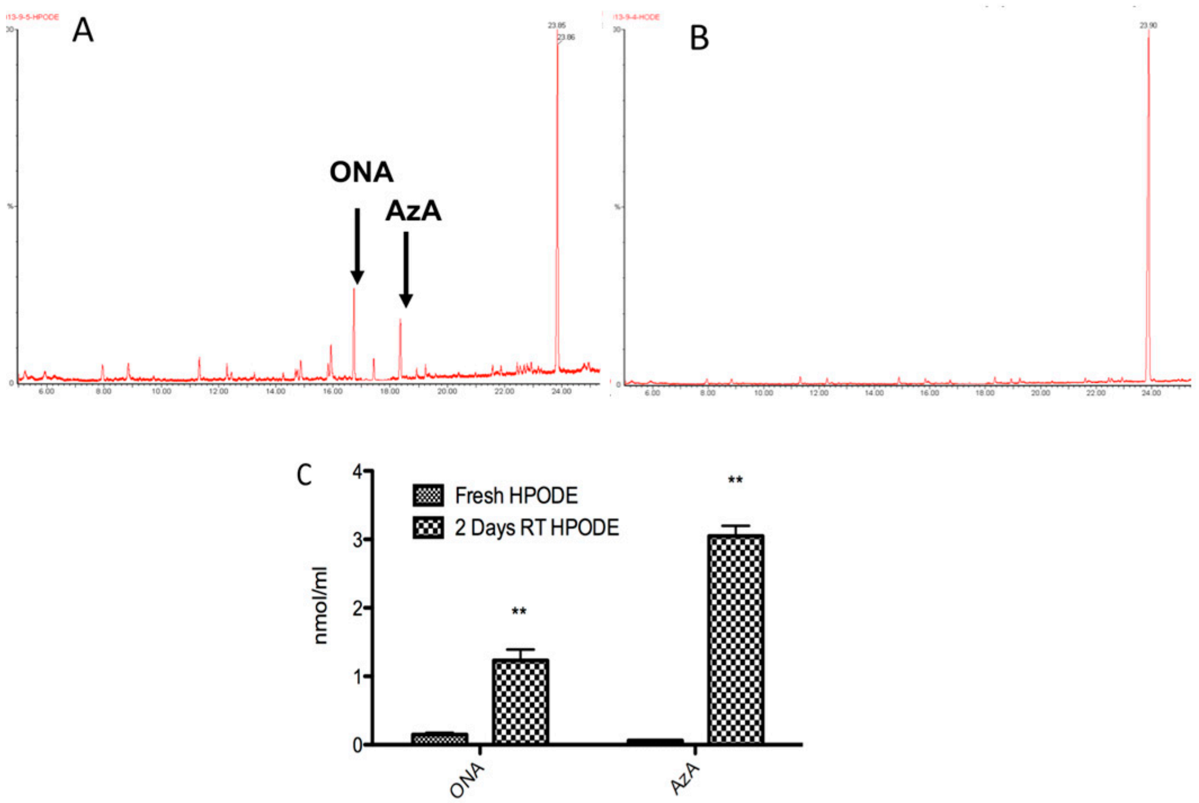

Figure S3. Decomposition products of 13-HPODE at room temperature for 0hr and 48hr. (A) Representative gas chromatogram of 13-HPODE at room temperature (RT) for 48hrs. **(B)** Representative gas chromatogram of freshly prepared 13-HPODE, and **(C)** GC-MS quantification of 9-ONA and AzA from samples of freshly prepared 13-HPODE and 13-HPODE incubated for 48hrs at room temperature (n = 9/group; ** $p < 0.01$).

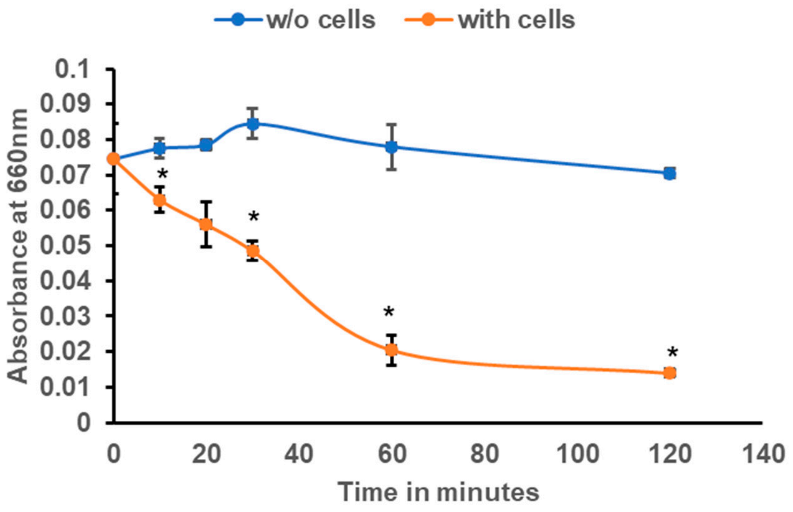

Figure S4. **Decomposition of 13-HPODE in the presence or absence of fully differentiated Caco-2 cells by LMB assay.**

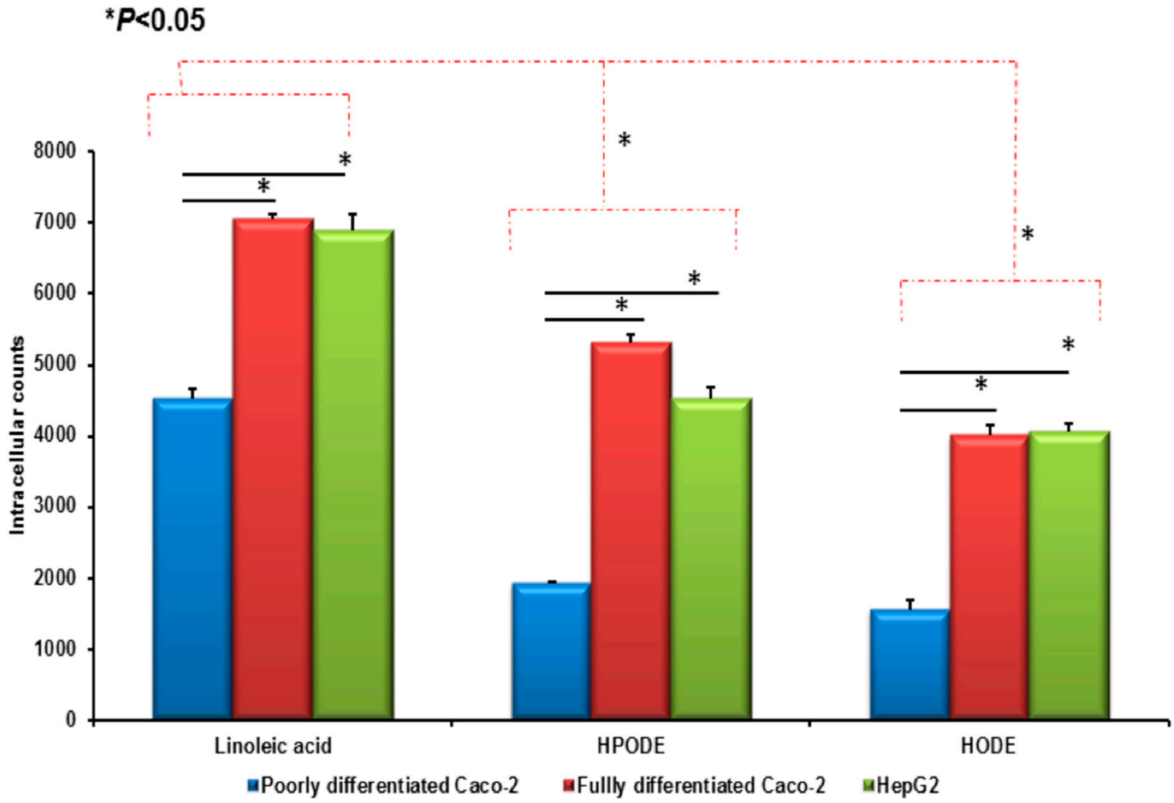

Figure S5. **Cellular uptake of linoleic acid and oxidized decomposition products. Uptake of ^{14}C-labeled LA, 13-HPODE, and 13-HODE by poorly differentiated Caco-2, fully differentiated Caco-2 and HepG2 cells was determined and adjusted to cellular protein concentration (n = 9/group; p < 0.05).**

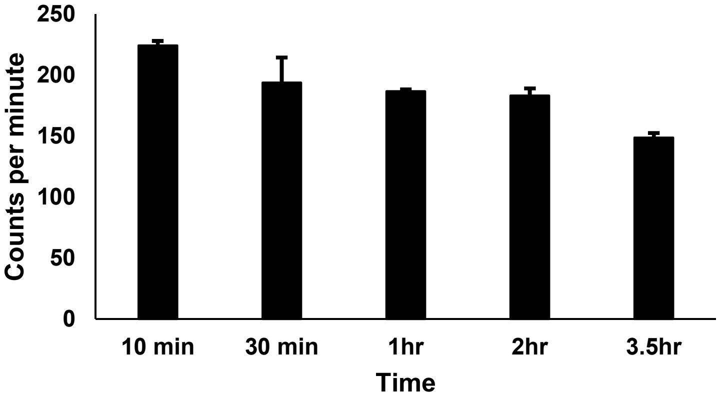

Figure S6. Quantification for ^{14}C-13-HPODE TLC counts over 3.5hrs.

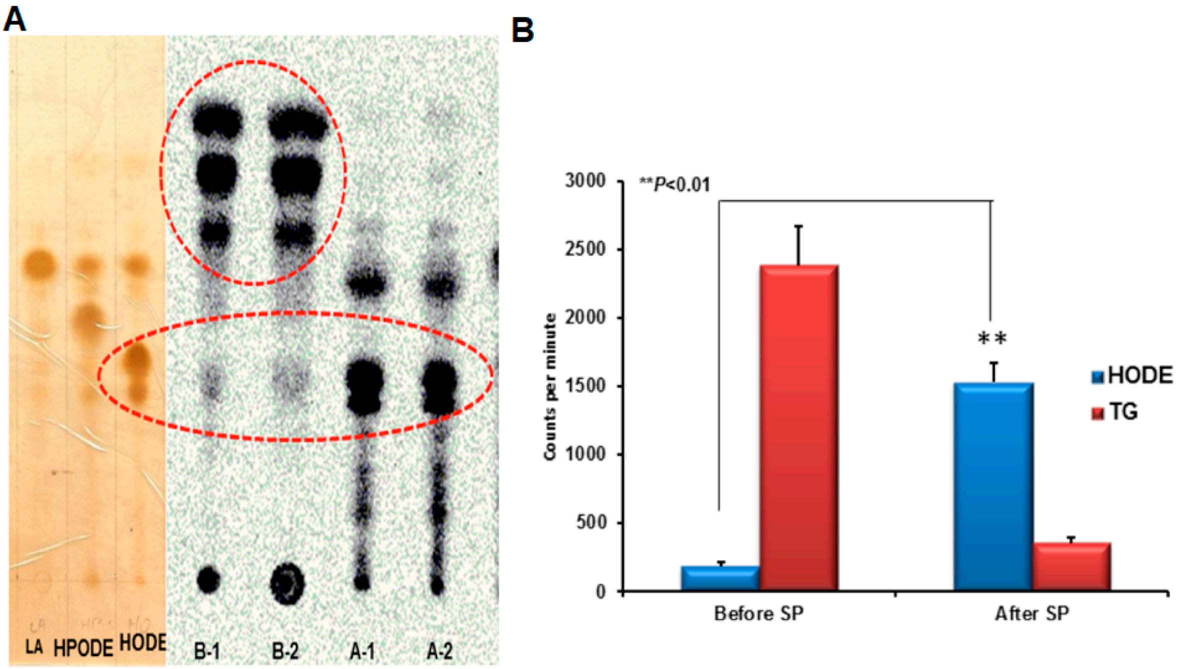

Figure S7. Representative TLC radioautograph of ^{14}C-13-HPODE and decomposition products from fully differentiated Caco-2 cells before and after saponification (SP). Panel **(A):** Intracellular lipid fractions before (B-1 and B-2 spots) and after (A-1 and A-2 spots) saponification (1 and 2 represent different samples). Panel **(B):** Quantification of radioactive counts of 13-HODE and triacylglycerol (TG) fractions on TLC plate (n = 9/group; ** $p < 0.01$).

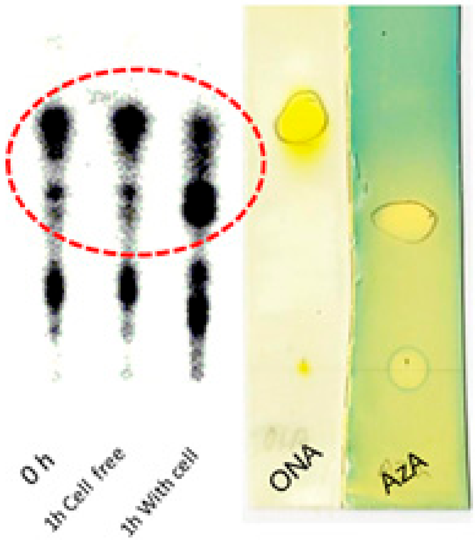

Figure S8. Representative TLC radioautography of media following ^{14}C-9-ONA treatment of fully differentiated Caco-2 cells or cell-free media for 1hr.

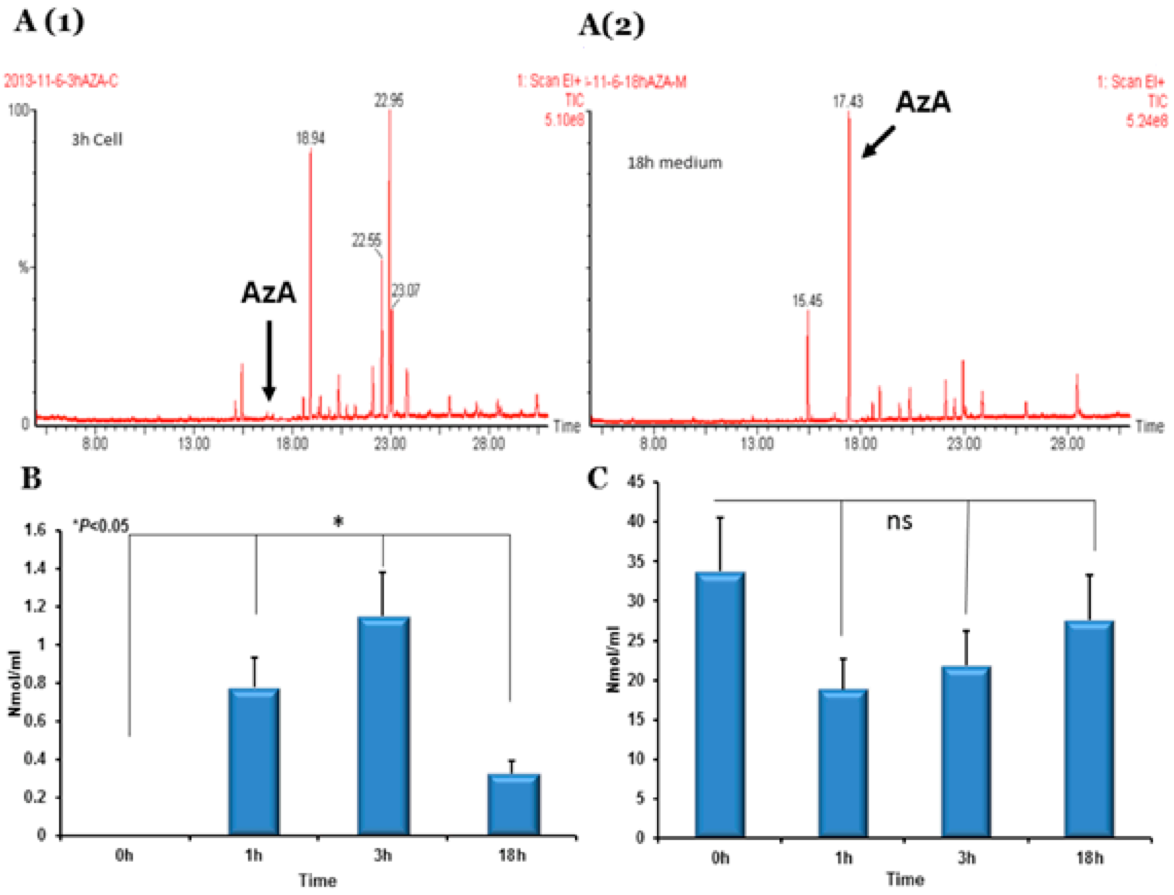

Figure S9. AzA remains detectable in fully differentiated Caco-2 cell culture media. (A1-2) Representative gas chromatograms of lipid extract from AzA-treated Caco-2 cell lysate and media, respectively. **(B)** Quantification of intracellular AzA over 18hrs following saponification ($n = 3$; $*p < 0.05$). **(C)** Quantification of cell media AzA over 18hrs ($n = 3$). Data pooled from 3 independent experiments.

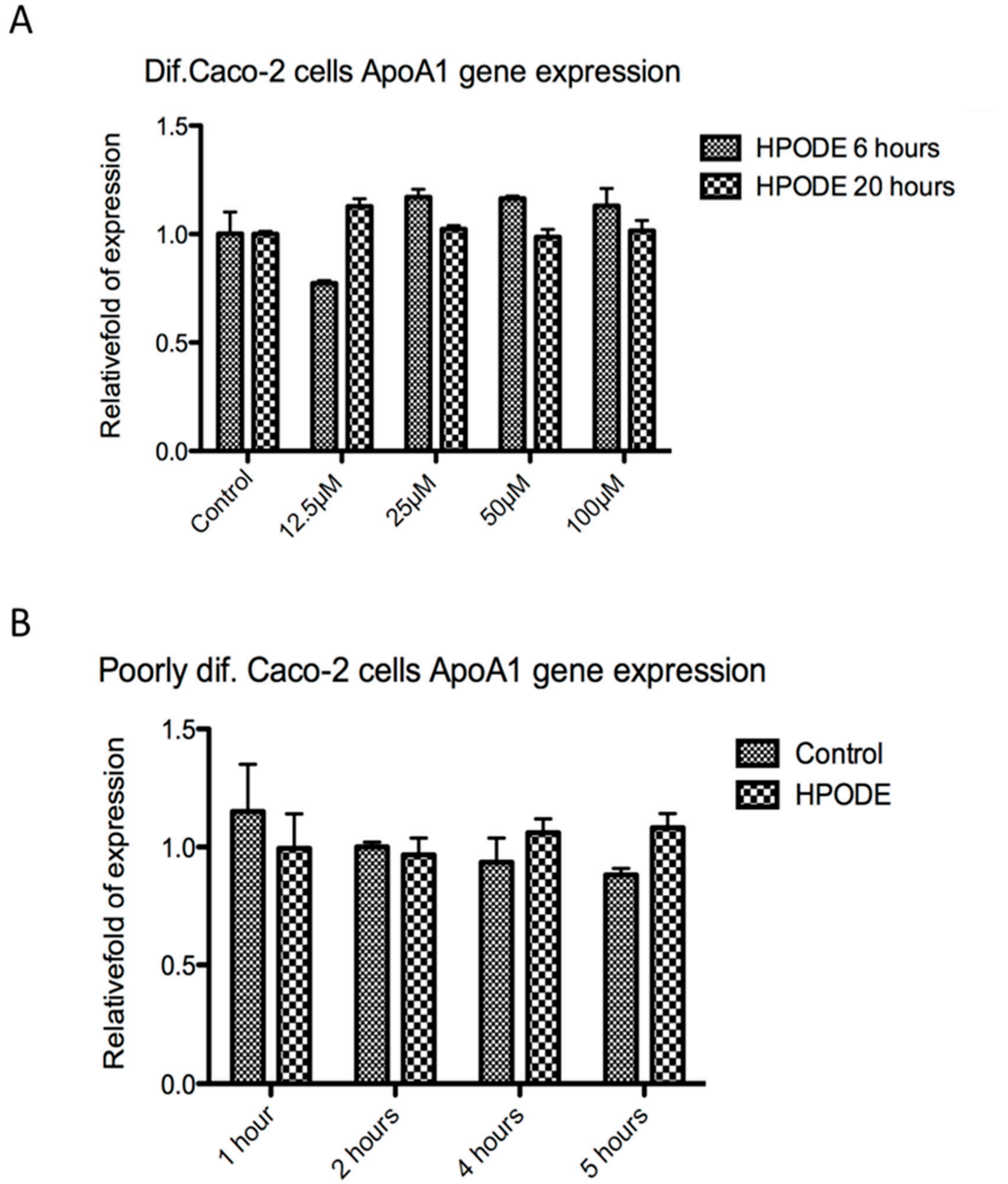

Figure S10. Normalized gene expression of ApoA1 in Caco-2 cells following 13-HPODE treatment. (A) Fully differentiated Caco-2 cells with 13-HPODE concentrations from 0-100μM and **(B**) poorly differentiated Caco-2 cells in comparison to control (no 13-HPODE) (*n* =6/treatment).

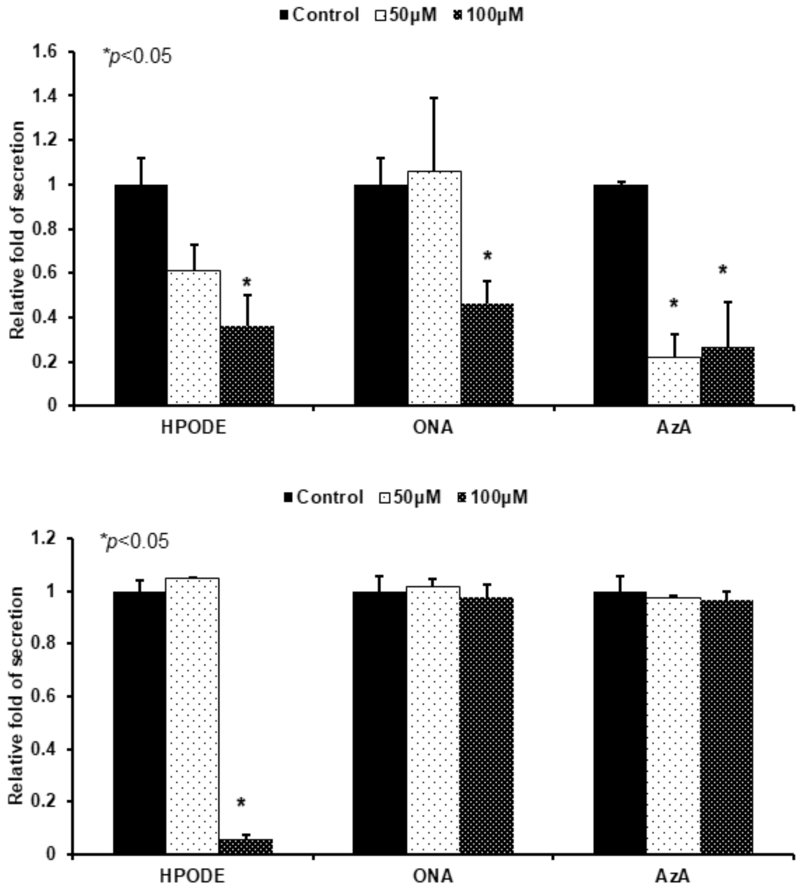

Figure S11. ApoB secretion following 24hr 13-HPODE, 9-ONA, and AzA treatment. (A) Fully differentiated Caco-2 cell media and **(B)** HepG2 cell media ($n \geq 9$/group, *$p < 0.05$).
